# 

**Published:** 1886-02

**Authors:** 


**Cutaneous Memoranda.** By HENRY G. PIFFARD, A. M., M. D., Clinical Prof. of Dermatology, University City of New York. Third edition. New York: Wm. Wood & Co., 56 Lafayette Place. 1885.

